# Comprehensive Analysis of Lutein and Loroxanthin in *Scenedesmus obliquus*: From Quantification to Isolation

**DOI:** 10.3390/molecules29061228

**Published:** 2024-03-09

**Authors:** Ayşegül Erdoğan, Ayça Büşra Karataş, Dilan Demir, Zeliha Demirel, Merve Aktürk, Öykü Çopur, Ali Çağır, Meltem Conk-Dalay

**Affiliations:** 1 Application and Research Centre for Testing and Analysis (EGE MATAL), Ege University, 35100 İzmir, Türkiye; 2 Department of Bioengineering, Faculty of Engineering, Ege University, 35100 İzmir, Türkiye; ayca.b.karatas91@gmail.com (A.B.K.); dilanndemirr@gmail.com (D.D.); zelihademirel@gmail.com (Z.D.); oykucopr@gmail.com (Ö.Ç.); meltemconkdalay@gmail.com (M.C.-D.); 3 Department of Chemistry, Faculty of Science, Ege University, 35100 İzmir, Türkiye; akturkmerve90@gmail.com; 4 Department of Chemistry, Faculty of Science, İzmir Institute of Technology, 35430 İzmir, Türkiye; alicagir@iyte.edu.tr

**Keywords:** *Scenedesmus obliquus*, carotenogenesis, extraction, lutein, loroxanthin, nitrogen sources

## Abstract

Carotenoids are hydrophobic pigments produced exclusively by plants, fungi, and specific microbes. Microalgae are well suited for the production of valuable carotenoids due to their rapid growth, efficient isoprenoid production pathway, and ability to store these compounds within their cells. The possible markets for bio-products range from feed additives in aquaculture and agriculture to pharmaceutical uses. The production of carotenoids in microalgae is affected by several environmental conditions, which can be utilized to enhance productivity. The current study focused on optimizing the extraction parameters (time, temperature, and extraction number) to maximize the yield of carotenoids. Additionally, the impact of various nitrogen sources (ammonia, nitrate, nitrite, and urea) on the production of lutein and loroxanthin in *Scenedesmus obliquus* was examined. To isolate the carotenoids, 0.20 g of biomass was added to 0.20 g of CaCO_3_ and 10.0 mL of ethanol solution containing 0.01% (*w*/*v*) pyrogallol. Subsequently, the extraction was performed using an ultrasonic bath for a duration of 10 min at a temperature of 30 °C. This was followed by a four-hour saponification process using a 10% methanolic KOH solution. The concentration of lutein and loroxanthin was measured using HPLC–DAD at 446 nm, with a flow rate of 1.0 mL/min using a Waters YMC C_30_ Carotenoid column (4.6 × 250 mm, 5 μm). The confirmation of carotenoids after their isolation using preparative chromatography was achieved using liquid chromatography–tandem mass spectrometry (LC–MS/MS) with an atmospheric pressure chemical ionization (APCI) probe and UV–vis spectroscopy. In summary, *S. obliquus* shows significant promise for the large-scale extraction of lutein and loroxanthin. The findings of this study provide strong support for the application of this technology to other species.

## 1. Introduction

Carotenogenesis, the biosynthetic process leading to carotenoid formation, is a critical pathway in many microalgae [1,2,3,4]. Carotenoids, such as loroxanthin and lutein, are essential pigments with a significant commercial and health-related value, due to their antioxidant properties and roles in human nutrition [5,6]. Understanding the factors that influence carotenoid biosynthesis in green microalgae is vital for optimizing their production, extraction, and isolation for commercial purposes. Carotenoid production in green microalgae represents a significant area of interest in biotechnological research due to its wide range of applications in food, cosmetic, and pharmaceutical industries [7,8,9,10]. Carotenoids, the naturally occurring pigments synthesized by plants, algae, and photosynthetic bacteria, are crucial for photosynthesis and photoprotection. In green microalgae, these pigments play a key role in adapting to varying environmental conditions [11,12]. Among various factors influencing carotenoid synthesis, the type of nitrogen sources available and their concentrations in growth medium are of importance [13].

Nitrogen, a key nutrient in algal growth media, significantly impacts metabolic pathways including those involved in carotenoid biosynthesis. The nature of the nitrogen source—whether ammonia, nitrite, nitrate, urea, or other organic nitrogen forms—can lead to variations in carotenoid content and composition This is attributed to the nitrogen source’s role in enzyme activity regulation within the carotenogenic pathway, altering the metabolic flux between primary and secondary metabolites. Research in this area often focuses on optimizing nitrogen levels and sources to enhance carotenoid productivity in green microalgae. Studies have shown that nitrogen deprivation or stress can trigger an increase in carotenoid concentration, particularly those with antioxidant properties such as lutein, β-carotene, and astaxanthin [14,15,16,17]. However, the underlying molecular mechanisms and metabolic responses to different nitrogen sources vary among microalgal species, making it a complex area of study.

Furthermore, understanding the effect of nitrogen sources on carotenoid production is not only crucial for enhancing yield but also for controlling the quality of the carotenoids produced. This knowledge aids in the development of sustainable and economically viable methods for the large-scale production of valuable carotenoids from green microalgae. In the literature, research mostly focuses on the impact of nitrogen deprivation or limitation on the biomass growth and the accumulation of carotenoids. The production of carotenoids in green microalgae is significantly influenced by the type and availability of nitrogen sources. Nitrogen is a critical nutrient in algal growth and metabolic processes, including the synthesis of carotenoids [18,19,20]. Studies have shown that nitrogen-rich conditions generally promote higher biomass productivity, which can lead to increased carotenoid synthesis. For instance, microalgae like *Nephroselmis* sp. exhibits enhanced carotenoid biosynthesis under nitrogen-replete conditions, correlating with a higher antioxidant capacity [21]. However, the relationship between nitrogen availability and carotenoid content is not linear or uniform across all microalgal species. Some species might increase carotenoid production as a stress response under nitrogen limitation or starvation. This variable response can be attributed to the activation of different metabolic pathways under stress conditions, where carotenoids serve protective roles against oxidative damage and environmental stressors [22].

Moreover, the type of nitrogen source also plays a crucial role. Nitrate (NO_3_^−^) and ammonium (NH_4_^+^) are commonly used nitrogen sources in microalgal cultures. While nitrate is a stable nitrogen source, its assimilation requires more energy due to the reduction steps involved in converting nitrate to ammonia before it can be incorporated into organic molecules [23]. On the other hand, ammonium can be directly assimilated; but at high concentrations, it can be toxic to some microalgal species [24]. Attractively, some studies suggest that using nitrite (NO_2_^−^) as a nitrogen source can be advantageous, potentially due to its reduced form, which requires fewer steps for assimilation compared to nitrate, thus being more energy efficient. However, the impact of nitrite on carotenoid production specifically requires more focused research. In summary, the quantity and type of nitrogen source are critical factors in modulating carotenoid production in green microalgae, with different species responding uniquely to variations in nitrogen conditions [15,16,25,26].

This research employed controlled experimental setups where cultures of *S. obliquus* were grown in media supplemented with various nitrogen sources. The concentrations of loroxanthin and lutein were monitored and analyzed for each biomass. After achieving optimal growth and carotenoid accumulation, preparative chromatography techniques were used to isolate and purify these carotenoids. This research focused on how different nitrogen sources affect loroxanthin and lutein production in *S. obliquus*, before the optimization of their extraction parameters to enhance the carotenoid production and their isolation using preparative chromatography. The findings from this study have the potential to significantly impact the commercial production of carotenoids. By identifying the optimal extraction parameters and nitrogen source for loroxanthin and lutein production in *S. obliquus*, this research could lead to more efficient and cost-effective methods for producing these valuable compounds. Additionally, understanding the metabolic responses of *S. obliquus* to different nitrogen sources can provide insights into the broader field of algal biotechnology and biochemistry, particularly in the area of metabolic engineering for enhanced secondary metabolite production.

## 2. Results

### 2.1. Optimization of Extraction Parameters for the Carotenoid Production in S. obliquus

In the present study, ultrasound-assisted extraction was employed. The extraction parameters were selected as the time, temperature, and the number of extractions which affected the lutein and loroxanthin content. Based on the results, a 10 min extraction at 30 °C was enough to achieve the maximum amount of both carotenoids. In addition, three successive extraction procedures were sufficient to remove almost all lutein and loroxanthin from *S. oblquus.*

#### 2.1.1. Effect of Temperature on the Extraction of LU and LX

**Figure 1:**
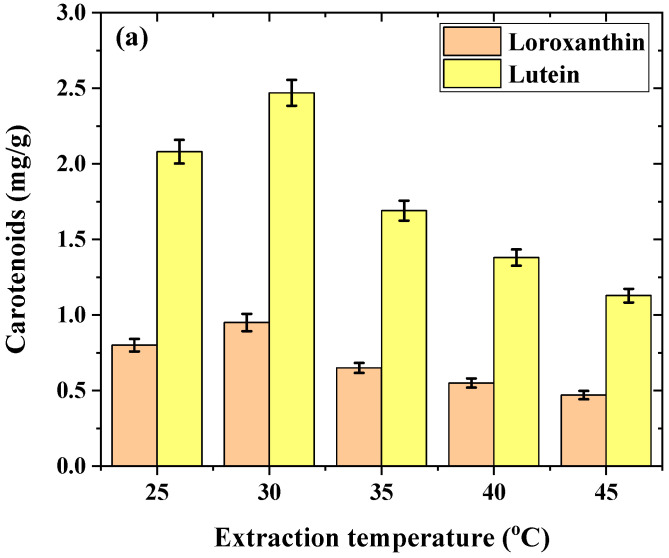

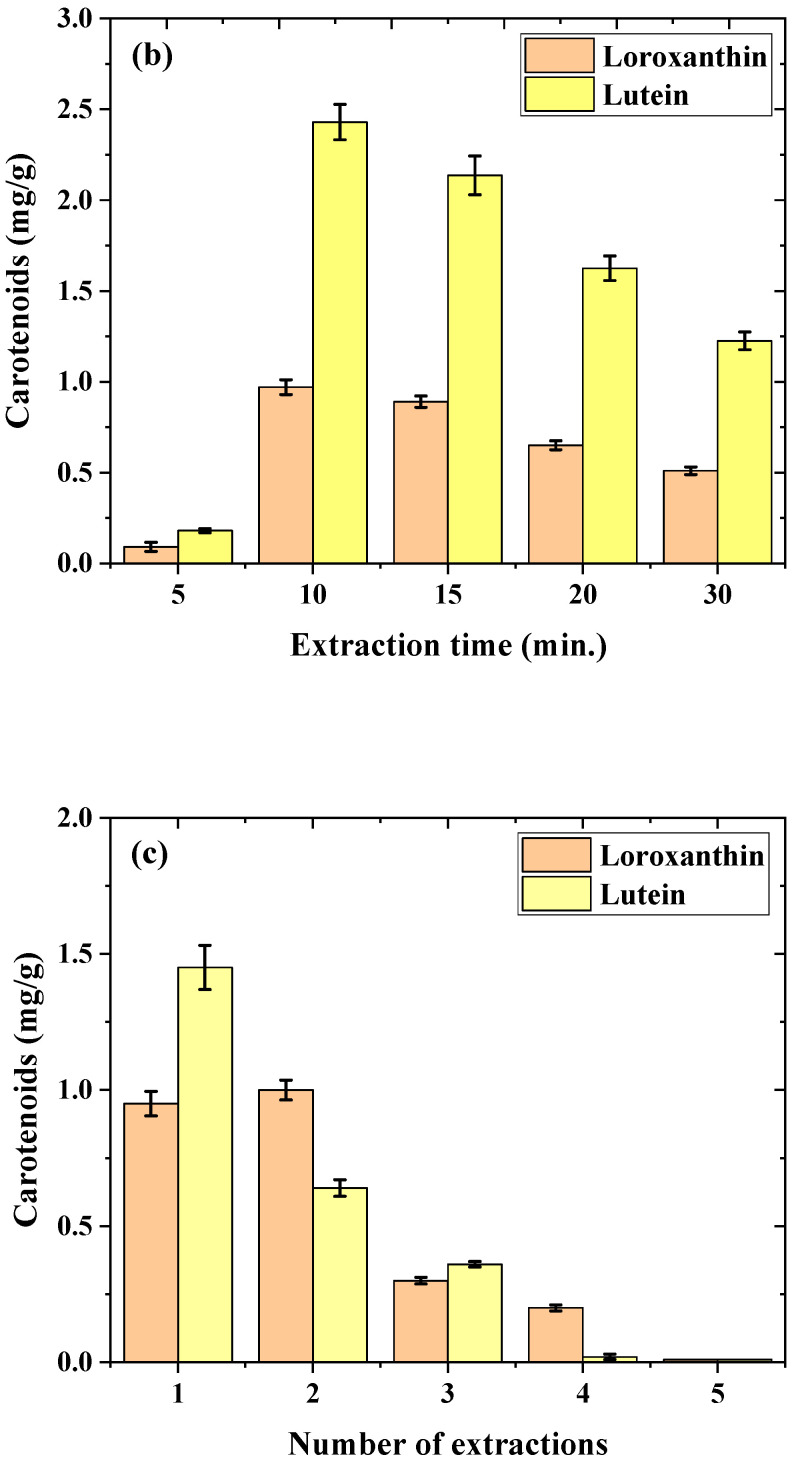
Optimization of extraction parameters on lutein and loroxanthin content. (**a**) Effect of temperature. (**b**) Effect of time. (**c**) Effect of extraction number. Experimental conditions: 10 mL ethanol, 0.25 g biomass, *n* = 3.

Temperature is an important factor affecting extraction. While moderate heat can enhance solubility and diffusion rates, thus initially improving extraction efficiency, excessive temperatures often lead to the thermal degradation of carotenoids [27]. These pigments are susceptible to isomerization and oxidation at high temperatures, which not only reduces their overall yield but also affects their quality and nutritional value. It is essential to select the optimum temperature to achieve a high extraction rate; hence, the influence of temperature on the quantities of extracted loroxanthin and lutein was also investigated. The findings of this study are displayed in Figure 1a. The extraction temperatures were adjusted to 25, 30, 35, 40, and 45 °C. 

#### 2.1.2. Effect of Time on the Extraction of LU and LX

The duration of extraction is another crucial factor in solvent extraction. The fundamental mechanism involves the transfer of carotenoids from microalgae to the extraction solvent by diffusion and penetration [28]. The relationship between the time spent on the extraction process and the amount of extracted content is illustrated in Figure 1b. The ultrasonic extraction duration varied between 5, 10, 15, 20, and 30 min, at a temperature of 30 °C. 

#### 2.1.3. Effect of the Number of Extractions on LU and LX Amounts

The number of extraction cycles significantly influences the amount of carotenoids extracted from microalgae. In general, multiple extraction steps can lead to a higher cumulative yield of carotenoids. In this study, the effect of the number of extractions on the loroxanthin and lutein yield is presented in Figure 1c. Based on the results, three extractions seemed to be enough for high carotenoid yields.

### 2.2. Effect of Nitrogen Sources on the Cell Growth and the Carotenoid Content of S. obliquus

It is important to note that the response of microalgae to different nitrogen sources can vary significantly between species and its impact on carotenoid production can depend on a multitude of factors including species-specific metabolism, culture conditions, and the presence of other nutrients [29,30]. Research in this area is ongoing and more specific studies are needed to fully understand the implications of using nitrite as a nitrogen source for carotenoid production in different microalgal species [31].

In our study, we observed an increase in the amount of carotenoids, whereas the specific growth rate was statistically not changed when nitrite was used as a nitrogen source instead of nitrate. The amount of lutein increased from 5.25 ± 0.08 mg/g to 11.08 ± 0.12 mg/g and the loroxanthin content increased from 2.40 ± 0.05 mg/g to 5.46 ± 0.11 mg/g, as presented in Table 1. On the other hand, biomass could not be obtained when urea and ammonium chloride were used during cultivation. Therefore, the extraction could not be performed. 

### 2.3. Isolation of Loroxanthin and Lutein Using Preparative Chromatography

**Figure 2:**
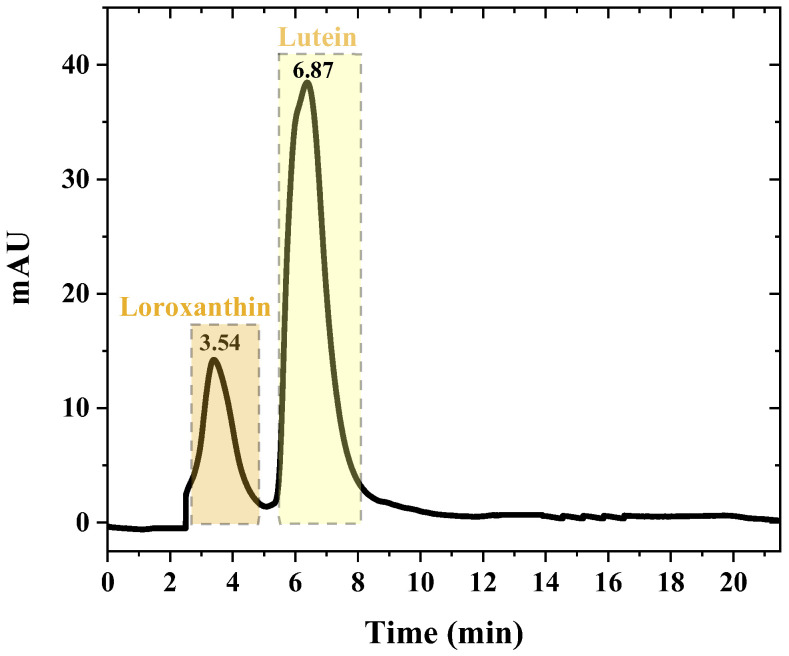
prep-HPLC chromatogram for *S. obliquus*.

Preparative chromatography can be tailored to target specific carotenoids by adjusting parameters such as the mobile phase composition, flow rate, and temperature. This method is highly valued for its precision and ability to yield high-purity carotenoids. To achieve the isolation of pure chemicals, the microalgae extract underwent prep-HPLC for subsequent purification. The choice of the prep-HPLC mobile phase was determined by considering the polarity of the two substances and the analytical HPLC conditions. A binary mobile phase system consisting of methanol and methyl-tert-butyl ether at a ratio of 85:15 was utilized, with a flow rate of 4.0 mL/min. The measurements were conducted at a wavelength of 450 nm. Given the parameters indicated above, a successful separation of each desired chemical was accomplished, as illustrated in Figure 2. The waste products of each specific drug were gathered and examined using HPLC–DAD and LC–APCI–MS/MS to identify them more accurately.

### 2.4. Identification of Loroxanthin and Lutein Using HPLC–DAD and LC–APCI–MS/MS

**Figure 3:**
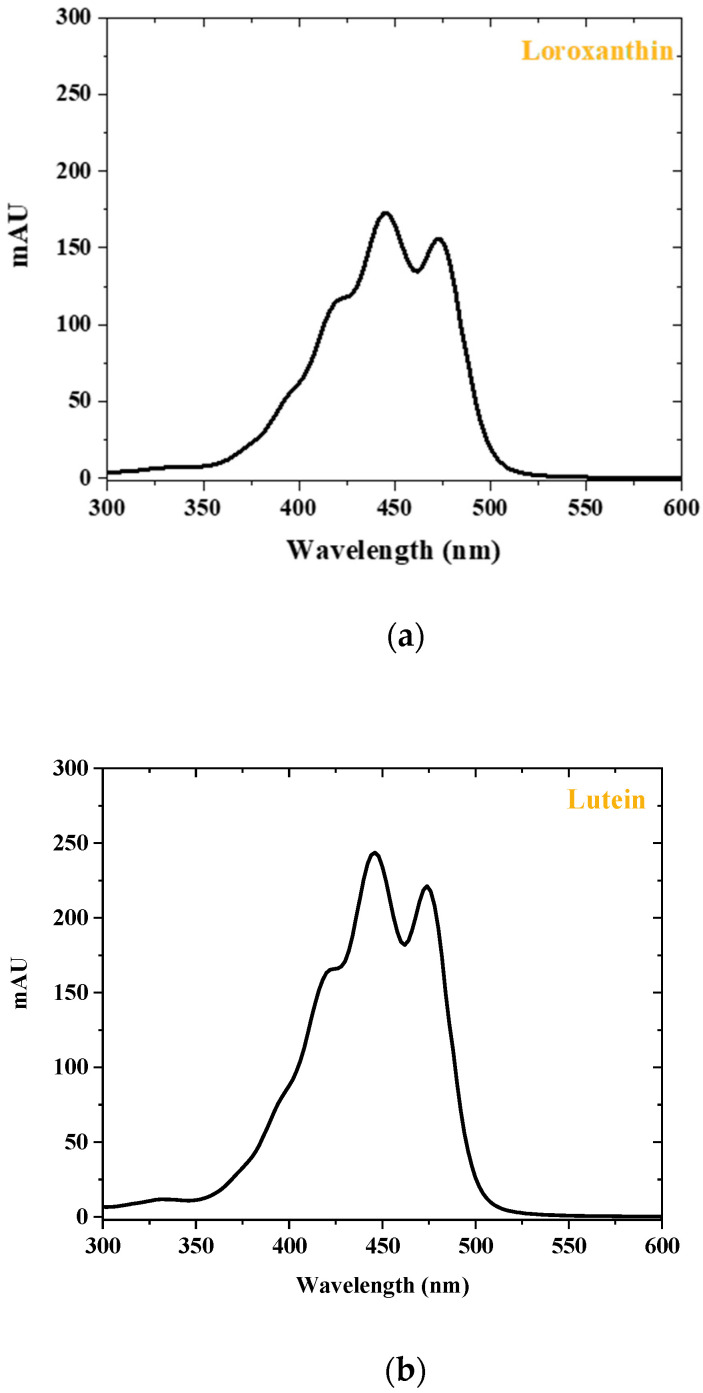
UV–visible spectrum for (**a**) loroxanthin and (**b**) lutein.

**Figure 4:**
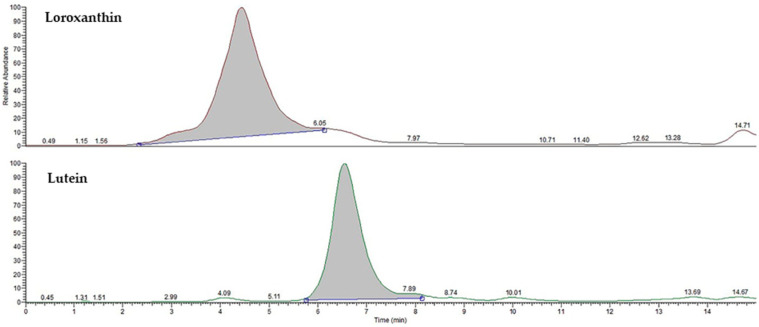
LC–MS chromatogram for isolated loroxanthin and lutein from *S. obliquus*.

**Figure 5:**
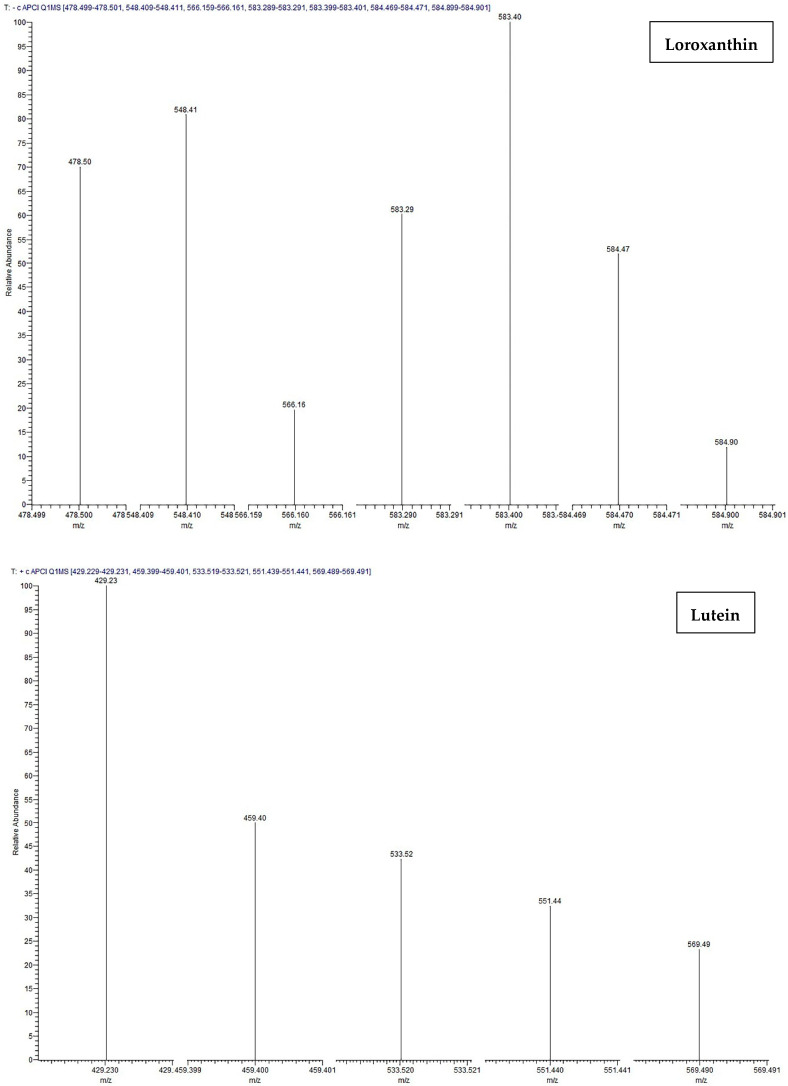

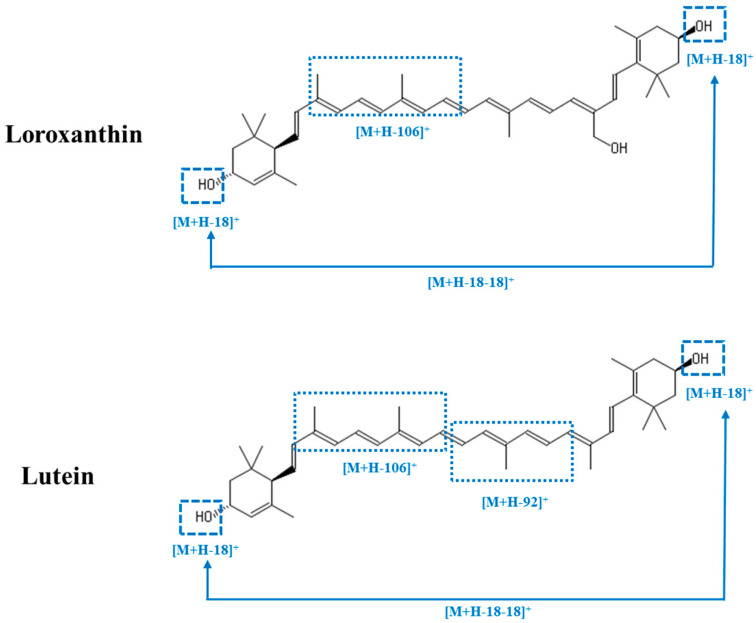
LC–MS/MS spectra of isolated loroxanthin and lutein and their possible fragmentation pathways.

Chromatography offers significant advantages, making it an ideal choice for researchers and industries focused on these valuable pigments. Preparative HPLC is highly efficient in separating carotenoids, which often coexist in complex mixtures with other compounds. This method is particularly beneficial for carotenoids due to their sensitivity to heat and light; HPLC operates under conditions that minimize degradation, ensuring the integrity of these delicate molecules. The versatility of HPLC also allows for the optimization of separation conditions tailored to different types of carotenoids, whether they are more polar or non-polar. This integrative approach enhances the overall efficiency and effectiveness of the isolation process. The investigation involved dissolving pure carotenoids in a mobile phase and then injecting them into an HPLC–DAD system. The HPLC–DAD analysis involved the collection of absorbance data within the wavelength range of 300 to 600 nm. Based on the acquired results (Figure 3), the fine structures and absorbance values of loroxanthin and lutein are consistent with the values reported in the literature. The fractions obtained after preparative chromatography were additionally diluted in 10.0 mL of methanol and analyzed using LC–MS/MS to determine the molecular weights of the carotenoids. The identification and confirmation of loroxanthin and lutein were achieved through the use of retention time (RT) and Selected Ion Monitoring (SIM) mode. The analysis was conducted in positive ion mode and optimized using a commercially available lutein standard. Since there is no available loroxanthin standard, the same conditions were used for its identification. Figure 4 illustrates the chromatogram of pure loroxanthin and lutein obtained from *S. obliquus*. LC–MS/MS spectra are shown in Figure 5, with the possible fragmentation pattern of loroxanthin and lutein. The presence of ions with m/z values of 584.47 [M]^+^, 584.90 [M + H]^+^, 583.40 [M + H-2]^+^, 566.16 [M + H-18]^+^, 548.41 [M + H-2-18-18]^+^, and 478.50 [M + H-106]^+^ confirms the purification of loroxanthin. Similarly, the ions with *m*/*z* values of 569.49 [M + H]^+^, 551.44 [M + H-18]^+^, 533.52 [M + H-18-18]^+^, 459.40 [M + H-92-18]^+^, and 429.23 [M + H-106-18-16]^+^ indicate the isolation of lutein, which aligns with the literature values [32].

## 3. Discussion

### 3.1. Influence of Extraction Conditions

The optimization of extraction parameters for enhanced carotenoid production in green microalgae is significant, due to the rising demand for natural carotenoids in various industries, including food, cosmetics, and pharmaceuticals [10,33,34]. Carotenoids are not only valued for their vibrant colors but also for their potent antioxidant properties, which play a critical role in human health by protecting cells from oxidative damage. Microalgae, as a sustainable and efficient source of carotenoids, have attracted considerable interest in biotechnological research [35,36,37]. However, the effective extraction of these valuable carotenoids from microalgal biomass remains a challenge. This is mainly due to the presence of rigid cell walls in microalgae, which block the release of carotenoids. The extraction process is further complicated by the diversity of carotenoids, each with unique solubility and stability characteristics. Therefore, optimizing the extraction parameters, such as the choice of solvent, temperature, time, and extraction method, is critical to maximizing the yield, purity, and bioavailability of these compounds, while maintaining their functional integrity [38]. In this context, recent advancements in extraction technologies and methodologies offer promising avenues for enhancing carotenoid recovery from green microalgae. Techniques such as atmospheric liquid extraction with maceration [39], pulsed electric field-assisted extraction [40], enzyme-assisted extraction [41], accelerated solvent extraction [42], microwave-assisted extraction [43], supercritical fluid extraction [44,45], ultrasonic-assisted extraction [30,40], and enzyme-assisted extraction [41] have shown potential in improving extraction efficiency compared to traditional methods. 

The levels of loroxanthin and lutein had a significant increase and approached their peak at a temperature of 30 °C during the ultrasonic extraction process lasting 10 min. The amount of carotenoids decreased with the increasing temperature. This effect was expected. The temperature of the rotary evaporator was also set to 40 °C for all evaporation processes, since carotenoids decompose at temperatures exceeding the chemical structure’s sensitivity to heat. The same situation is valid for the increased extraction temperature. This decomposition is primarily due to the instability of carotenoids’ long chains of carbon atoms with conjugated double bonds, which, under heat, break down, leading to the degradation of their molecular structure. Additionally, the presence of oxygen can accelerate this degradation through oxidative stress, causing radical formation that further attacks the carotenoid molecules. Heat also induces isomerization, a re-arrangement of the molecule’s structure, altering its physical and chemical properties. These processes result in the loss of color, antioxidant capacity, and the overall functionality of carotenoids, posing challenges in their application in food, pharmaceutical, and cosmetic industries, where their stability is crucial [46,47,48,49,50]. 

The greatest yields of loroxanthin and lutein were obtained after a 10 min extraction period. This might be due to the phenomenon of equilibrium and the potential degradation of target compounds. Initially, extraction is efficient because of the high concentration gradient between the compound in the material and the solvent. As extraction progresses, this gradient diminishes, and the system approaches an equilibrium where the rate of solute being extracted equals the rate of its re-absorption or adherence back to the solid matrix. Moreover, carotenoids are sensitive compounds; prolonged exposure to extraction conditions like light, heat, and oxygen can result in their degradation or isomerization [28,51]. 

The first extraction cycle usually removes the most accessible carotenoids, but not all are extracted due to limitations in solubility and diffusion through the cell matrix. Subsequent extractions can access more deeply embedded carotenoids, resulting in additional yields. However, there is often a diminishing return with each additional extraction; each successive cycle tends to extract less than the previous one, as the concentration of extractable carotenoids in the microalgae decreases [52]. It is also important to balance the benefits of increased yield against the additional time, solvent usage, and potential degradation risks associated with multiple extractions. Therefore, optimizing the number of extractions is crucial to efficiently maximize the yield of carotenoids from microalgae, while considering both the economic and practical aspects of the extraction process [53,54]. In the present study, the extraction of loroxanthin and lutein from *S. obliquus* was optimized. According to the results obtained, the maximum carotenoid amounts were achieved at 30 °C for a 10 min period, after three successive extractions. 

### 3.2. Influence of Different Nitrogen Sources

Different nitrogen sources, including ammonia, nitrate, nitrite, and urea, can be employed to enhance the growth of microalgae. In the typical nitrogen assimilation pathway in green microalgae, nitrate must first be reduced to nitrite and then to ammonia before it can be incorporated into amino acids and other nitrogen-containing organic molecules. This reduction process involves enzymes like nitrate reductase and nitrite reductase. The energy for these reduction steps is derived from ATP and reducing agents like NAD(P)H, which are generated through photosynthesis. Therefore, when nitrate is used, more energy is expended in these initial reduction steps.

The literature demonstrates that the selection of an appropriate nitrogen source is crucial for enhancing the biomass productivity of microalgal species [55]. Nevertheless, the impact of the nitrogen supply on the accumulation of lutein in microalgae remains uncertain. A study was conducted to evaluate the ideal nitrogen source for the growth of *S. obliquus* FSP-3 cells and the accumulation of lutein. Three distinct nitrogen sources, namely Ca(NO_3_)_2_, (NH_4_)_2_SO_4_, and urea, were studied at a concentration of 8.0 mM each [56]. The biomass production achieved by utilizing nitrate as a nitrogen source was approximately 2–3 times greater than that obtained from ammonium and urea. This suggests that nitrate is the preferred nitrogen source for the growth of S. obliquus FSP-3. This outcome was in line with findings derived from the growing of other species of microalgae. *Neochloris oleoabundans* exhibited superior development when nitrate was used as the nitrogen source, as opposed to ammonium and urea [57]. On the other hand, the microalgal species *Ellipsoidion* sp. exhibited a greater growth rate when exposed to ammonium [58]. Therefore, the appropriate nitrogen supply for the growth of microalgae is primarily determined by the species [55]. Microalgae typically prefer ammonium as a nitrogen source over nitrate since it does not involve a redox reaction and requires less energy for assimilation [59]. Nevertheless, the reduced biomass production seen in *S. obliquus* FSP-3 when exposed to ammonium can be attributed to a significant decline in pH, dropping from 7.0 to around 5.0, during the assimilation of ammonium ions. Furthermore, a study demonstrated comparable findings, indicating that *Monoraphidium* species’ SB2 utilization of ammonium led to a significant decline in pH from 8.0 to 4.4, over a 55-day cultivation period [60]. Conversely, urea undergoes dissociation in solution through the urea amino hydrolase pathway (mediated by the urease enzyme) to produce carbon dioxide (CO_2_) and ammonium ions [59]. The reduced biomass production observed with urea-N may be attributed to the comparatively poor urease activity of this strain.

In our study, it is evident that nitrite is the most advantageous nitrogen source for the stimulation of cell growth and the accumulation of carotenoids in *S. obliquus*, among four different nitrogen sources selected for cultivation. The idea that using nitrite (NO_2_^−^) as a nitrogen source can be advantageous for green microalgae, especially in the context of carotenoid production, stems from the understanding of nitrogen metabolism in these organisms. Nitrite, being in a more reduced form than nitrate (NO_3_^−^), requires fewer biochemical steps for assimilation into organic compounds, which can make it a more energy-efficient nitrogen source for microalgae [61]. When nitrite is provided as the nitrogen source, the step involving the reduction of nitrate to nitrite is bypassed, saving energy. This can be particularly advantageous under conditions where energy conservation is critical, such as under limited light or other stressful conditions. The more direct assimilation of nitrite can lead to a more efficient use of the microalgae’s metabolic resources, potentially channeling more energy towards growth and secondary metabolite production, including carotenoids. Moreover, carotenoids often play a role in the stress response of microalgae [62]. 

Urea is a small chemical compound that has a low molecular weight, exhibits polarity, and has a restricted ability to dissolve in lipids. It functions as a dual supplier of nitrogen and carbon. Several cases can be noticed in the literature where urea has demonstrated effectiveness as a source for the production of *Spirulina platensis* [63], *Neochloris oleoabundans* [57], and *Chlorella* sp. [64]. There has been previous research which took on the approach that urea was superior to nitrate, the typically utilized nitrogen source, in various aspects. Conversely, the growth rates of cultures cultivated with urea were inferior to the growth rates observed with alternative nitrogen sources. This disparity may arise due to the fact that urea is an organic nitrogen source, whereas the other substances are inorganic nitrogen sources [65]. Possibly, carotenoid accumulation due to the use of organic sources may not be the same when using inorganic sources, due to metabolic pathways. Moreover, this organic complex can be regarded as a combined source of both nitrogen and carbon. 

According to the findings of a number of researchers, ammonium is a great source of nitrogen for algae that live in both freshwater and marine environments [66,67,68,69]. On the other hand, it has not been utilized as extensively as nitrate as a source of nitrogen for the majority of algae, such as *Chlorella protothecoides* [70] and *Dunaliella salina* [71]. This may be because it is not suitable for sterilizing. 

In the present work, urea, and ammonium chloride, on the other hand, were found to be nitrogen sources that resulted in a high rate of cell mortality. This study showed that the consumption of ammonium and urea caused a significant decrease in pH and was the cause of the deadly effect on the cells. In the literature, it has been reported that the pH of a medium undergoes a significant shift as a culture develops, regardless of whether nitrate or ammonium is being utilized. The quick use of an ammonium ion by an alga, in particular, results in a significant decrease in pH and ultimately leads to the death of the culture [70]. It is probable that *S. obliquus* could not compensate for these pH fluctuations and the cells died. It may stem from the fact that urea’s ability to yield more at the same nitrogen content may be attributed to its tendency to undergo hydrolysis, resulting in the production of ammonia and bicarbonate [26]. Similarly, when ammonium was present in the medium, the sharp decrease in pH caused a fatal effect on the cells.

### 3.3. Loroxanthin and Lutein Content of Scenedesmus obliquus

The production and isolation of specific carotenoids from green microalgae hold considerable importance for several reasons, ranging from health benefits to environmental and industrial applications. Carotenoids are a class of natural pigments found in many plants and microorganisms, including green microalgae. They play essential roles in photosynthesis and protect cells from damage by absorbing blue light and acting as antioxidants. The interest in these compounds has grown significantly due to their potential in various sectors [72,73,74,75]. Sampathkumar and Gothandam studied the effect of sodium bicarbonate on lutein biosynthesis in *Chlorella pyrenoidosa*, highlighting the influence of environmental conditions on lutein production in green microalgae [76]. They found that the lutein content was induced (4.84 ± 0.56 mg/g of DCW). In addition, 6.71 mg/g lutein from *Parachlorella kessleri* HY1 strain was obtained in a study conducted by Fábryová et al., using maceration extraction [77]. Furthermore, *Scenedesmus almeriensis* has been identified as a potent producer of lutein, with a content of 8.54 mg/g, demonstrating a significant potential of certain green microalgae strains in producing high levels of lutein [78]. In another study performed with the same green microalga, the lutein content obtained was equal to 5.71 mg/g [79]. Erdoğan et al. studied different nitrogen sources in the cultivation of *Prochlorococus* sp. and found that the lutein content could increase to 3.34 mg when urea was used [26]. The carotenoid composition of *Scenedesmus protuberans* was also studied by Erdoğan et al., where the lutein amount was found to be 2.47 mg/g [80]. Finally, the content of lutein was reported to be 8.09 mg/g in *Desmodesmus* sp. In our study, the lutein amount could be increased to 11.08 mg/g by using nitrite as the nitrogen source while cultivating *S. obliquus*, which is a relatively high value. In addition, to the best of our knowledge, there has been no study to date on the determination or isolation of loroxanthin from green microalgae, so this study is the first and can be used as a comparison tool for future studies. In this work, loroxanthin could be obtained at 5.46 mg/g. This work holds significant value due to its investigation of the co-acquisition of lutein and loroxanthin. Given these circumstances, *S. obliquus* has demonstrated its potential as a feasible source for the production of both lutein and loroxanthin.

## 4. Materials and Methods

### 4.1. Chemicals and Reagents

All-trans-lutein, triethylamine, pyrogallol, potassium hydroxide, sodium sulphate, calcium chloride, and calcium carbonate were provided by Sigma-Aldrich (Darmstadt, Germany), and all the LC-grade solvents used in this study were purchased from Merck (Darmstadt, Germany).

### 4.2. Cultivation of S. obliquus Using Different Nitrogen Sources

In this study, green microalga *S. obliquus* was used and was supplied by Ege University Microalgae Culture Collection, EGEMACC-18 (https://ege-macc.ege.edu.tr/egemacc.com/cultures.php, accessed on 8 January 2024). First, cells were densified for the inoculum at 24 ± 2 °C and 100 µmol photons/m^2^s light intensity by gradually scaling up to a volume of 2 L. Afterwards, experiments were started by adding 10% of the inoculum. The experiments were carried out in a 1 L/min bubble photobioreactor at the same temperature and light intensity, with an aeration rate of 2 L/min. Throughout all productions, including the *S. obliquus* inoculum, we always used BBM (Bold’s Basal Medium) as the culture medium [81] (https://egemacc.com/cultures.php, accessed on 8 January 2024). In all productions, including the obliquus inoculum, BBM was always used as the culture medium and the cultivation lasted for 16 days. However, in the bubble photobioreactor productions in question, the media content was modified by changing the nitrogen sources, while keeping the nitrogen amount constant. NaNO_3_ was used as a control group with the standard nitrogen source of the BBM medium. Other selected nitrogen sources were NaNO_2_, NH_4_Cl, and CH_4_N_2_O [82,83].

### 4.3. Determination of Growth Rate and Biomass Productivity 

The growth of *S. obliquus* was monitored by measuring the optical density at 680 nm at 2-day intervals, over a period of 16 days. The specific growth rate for green microalga was calculated according to Becker [84] (1994), using the data obtained by the absorbance values taken at 680 nm (Equation (1)).

(1) $$µ=\frac{\ln x_{2}-lnx_{1}}{\Delta t}$$

where μ = specific growth rate, *x*_2_ = cell concentration at time *t*_2_, *x*_1_ = cell concentration at time *t*_1_, and Δ*t* = *t*_2_ − *t*_1_. Doubling time was determined as 0.693/μ. 

### 4.4. Extraction and Saponification of Loroxanthin and Lutein from S. obliquus 

The carotenoid extract was obtained using a procedure that had been previously validated [85]. Concisely, 0.20 g of dehydrated biomass was measured and combined with 0.20 g of CaCO_3_. The mixture underwent extraction in an ultrasonic bath (Elmasonic S80H, Elma Schmidbauer GmbH, Singen, Germany) for 10 min at a temperature of 30 °C, utilizing 10.0 mL of ethanol solution containing 0.01% pyrogallol. The solution was subjected to centrifugation at a speed of 5000 revolutions per minute for two minutes. The liquid portion was kept, and the remaining solid was subjected to further extraction using fresh ethanol until the biomass became colorless. The combined solutions underwent additional vacuum filtration using a 47 mm diameter nylon filter paper (Sartorius) with a pore size of 0.20 µm. Subsequently, a 10% solution of methanolic KOH was introduced into the extract for saponification. This method lasts four hours, during which the maximum concentration of lutein is achieved (data not displayed). To stop the saponification process, a 10.0 mL solution of Na_2_SO_4_ at a concentration of 10% (*w*/*v*) was introduced. Subsequently, a volume of 10.0 mL of diethyl ether was introduced into the extract to combine the carotenoid fraction. The yellow-orange phase was collected multiple times until it became colorless. Subsequently, CaCl_2_ was introduced into the resulting solution of any residual water. The solution was, thereafter, subjected to rotary evaporation at a temperature of 40 °C and a pressure of 400 millibars using a Stuart RE 400 apparatus. 

Before HPLC analysis, the remaining substance was dissolved in 5.0 mL of chloroform that was fortified with 1% ethanol and then kept at a temperature of −20 °C. Each experiment was conducted in triplicate.

### 4.5. HPLC–DAD, Preparative HPLC and LC–MS/MS Analyses of Loroxanthin and Lutein

The concentration of loroxanthin and lutein in the carotenoid extract was measured by diluting it with a mobile phase consisting of methanol, methyl-tert-butyl ether, and water in a ratio of 70:25:5. Subsequently, 20 μL of the diluted extract was injected into an HPLC system (1260 Series, Agilent, Santa Clara, CA, USA) equipped with a diode array detector. The carotenoid extract was separated using a Waters YMC-C_30_ Carotenoid column (4.6 mm ID × 250 mm, 5 μm) at a flow rate of 1.0 mL/min and a temperature of 25 °C. The elution was monitored at wavelengths of 450 nm and 446 nm, which correspond to the maximum absorbance for loroxanthin and lutein, respectively. A gradient mobile phase system including methanol: methyl-tert-butyl ether: water was employed, as per prior research [26,80]. Based on the previous work, the percentage of water was added to methanol. To prepare a stock solution of lutein with a concentration of 100.0 mg/L, 1.0 mg of lutein was measured and dissolved in 10.0 mL of chloroform, which was stabilized with 1% ethanol. The quantification of loroxanthin (19-hydroxy lutein) and lutein was performed using High-Performance Liquid Chromatography with Diode Array Detection (HPLC–DAD). This was carried out by employing the external standard approach, where standard curves of a reference lutein standard were constructed. As there was no commercial loroxanthin standard available, the quantification of loroxanthin was performed using the standard curves of lutein, as both compounds have comparable structure and absorption spectra.

The separation of loroxanthin and lutein was conducted using the Thermo Fisher Scientific (Waltham, MA, USA) Ultimate 3000 HPLC system equipped with a YMC-C_30_ semi-prep Carotenoid column (10 mm ID × 250 mm, 5 μm). The mobile phase system utilized a binary gradient system, operating at a flow rate of 4.0 mL/min. The detection wavelength was set at 450 nm and the column temperature was maintained at 25 °C. The injection volume for the sample was 2.0 mL [85]. The gradient mobile phase was as follows: the fractions collected for each carotenoid were, thereafter, pooled and divided into two portions before their evaporation under nitrogen gas. Each substance was solubilized in methanol for subsequent LC–MS/MS analysis.

The LC–MS/MS system (Thermo Scientific/TSQ Quantum Access Max) with an atmospheric pressure chemical ionization (APCI) source was used to analyze and identify loroxanthin and lutein. The extract was fractionated using a C_30_ analytical column (4.6 mm inner diameter × 25 cm length, 5 μm particle size, YMC, Waters) at a temperature of 25 °C. The mass spectrometer was utilized in full scan mode, covering a range of *m*/*z* 50–900, with a vaporization temperature of 350 °C. The volume of the injection was 20 μL and the flow rate was 1.0 mL per minute. The mobile phase comprised methanol (A), an aqueous solution containing 0.1% formic acid (B), and tert-butyl methyl ether (C). The gradient elution was conducted according to the following protocol: from 0 to 5 min, a linear increase from 5% B to 25% C; from 5 to 10 min, a linear decrease from 5% B to 0% B and an increase from 35% C to 55% C; from 10 to 15 min, a constant concentration of 100% B; and a linear decrease from 0% B to 0% B and an increase from 55% C to 75% C. The mass spectra were obtained using positive ion mode, with a detection voltage of 1.5 kV and an APCI temperature of 350 °C. The identification of carotenoids was achieved through the comparison of their retention periods and mass spectra with those of reference standards and the mass spectra published in the literature [32,85,86].

### 4.6. Statistical Analysis

The experiment was conducted three times. Tukey’s test was employed at a significance level (*p* < 0.05) to identify variances among the treatment levels. The presented figures represent the average ± variability of the three measurements. The data were subjected to one-way analysis of variance (ANOVA) using Minitab (V18, Minitab Inc., State College, PA, USA). 

## 5. Conclusions

Microalgal biotechnology is a rapidly evolving field that capitalizes on the unique abilities of microalgae to produce valuable compounds, including carotenoids, under various culture conditions. The amount of carotenoids produced by microalgae can be significantly influenced by these conditions, which encompass light intensity, temperature, pH, nutrient availability, and the composition of the growth medium. Nutrient availability, particularly nitrogen, can modulate metabolic pathways towards enhanced carotenoid accumulation. By optimizing these culture conditions, researchers can maximize the yield of carotenoids, which are highly sought after for their antioxidant properties and applications in food, cosmetic, and pharmaceutical industries. This research offered practical applications in the field of microalgal biotechnology, particularly in the efficient extraction, enhanced productivity, and isolation of valuable carotenoids such as lutein and loroxanthin from *S. obliquus*. After the optimization of extraction conditions, the ultrasound-assisted extraction process was performed three times for 10 min at 30 °C and the amount of carotenoids reached their maximum values. Nitrite was found to be the best nitrogen source as the loroxanthin and lutein content were found to be 5.46 mg/g and 11.08 mg/g, respectively. Finally, these carotenoids could be isolated using preparative chromatography and identified with UV–visible spectroscopy and LC–MS/MS techniques. These attempts have shown that *S. obliquus* is applicable to the industrial production of both loroxanthin and lutein across different fields. In this sense, this study will shed light on future studies using different microalgae for the production and isolation of carotenoids.

## Figures and Tables

**Table 1:** Effect of different nitrogen sources on biomass productivity of *S. obliquus*, and loroxanthin and lutein contents.

Nitrogen Sources	Lutein	Loroxanthin	Specific Growth Rate
Containing Equivalent N Conc.	(mg/g)	(mg/g)	(µ)
NaNO_3_	5.25 ± 0.08 ^a^	2.40 ± 0.05 ^a^	0.303 ± 0.04 ^a^
NaNO_2_	11.08± 0.12 ^b^	5.46 ± 0.11 ^b^	0.322 ± 0.03 ^a^
CH_4_N_2_O	-	-	-
NH_4_Cl	-	-	-

Values for each stage within the same column bearing different superscripts are significantly different (*p* < 0.05), *n* = 3.

## Data Availability

All data generated or analyzed during this study are included in the article.
